# Correction of marked sagittal deformity with circumferential minimally invasive surgery using oblique lateral interbody fusion in adult spinal deformity

**DOI:** 10.1186/s13018-020-1545-7

**Published:** 2020-01-15

**Authors:** Seung Won Park, Myeong Jin Ko, Young Baeg Kim, Jean Charles Le Huec

**Affiliations:** 1 Department of Neurosurgery, College of Medicine, Chung-Ang University, 102 Heukseok-ro, Dongjak-gu, Seoul, 06973 Republic of Korea; 2 Head Department of Orthopedic surgery, Polyclinique Bordeaux Nord Aquitaine, 15-35 Rue Claude Boucher, 33300 Bordeaux, France

**Keywords:** Adult spinal deformity, Marked sagittal deformity, Sagittal correction, Minimally invasive spine surgery, Oblique lateral interbody fusion, Percutaneous fixation

## Abstract

**Background:**

Spinal surgery performed entirely with minimally invasive surgery is referred to as circumferential MIS (cMIS). However, cMIS still has a limited sagittal correction capability for adult spinal deformity (ASD) with a marked sagittal deformity. We investigated the effectiveness of cMIS using oblique lateral interbody fusion (OLIF) and percutaneous posterior spine fixation in correcting marked sagittal deformity.

**Methods:**

This study retrospectively evaluated 23 patients with ASD with marked sagittal deformity who underwent cMIS using OLIF without osteotomy and were followed-up for at least 24 months (whole group). The whole group was divided into the following two groups according to the type of interbody fusion at L5–S1: the OLIF51 group (*n* = 13) underwent OLIF at L1–L5 and L5–S1 and the TLIF51 group (*n* = 10) underwent OLIF at L1–L5 and transforaminal lumbar interbody fusion (TLIF) at L5–S1.

**Results:**

Sagittal vertebral axis (SVA; 125.7 vs. 29.5 mm, *p* < 0.001), lumbar lordosis (LL; 18.2° vs. 51.7°, *p* < 0.001), and pelvic incidence-LL mismatch (PI-LL, 35.5° vs. 5.3°) significantly improved postoperatively in the whole group. The OLIF51 group showed significantly higher postoperative LL than the TLIF51 group (55.5° vs. 46.9°, *p* < 0.001). OLIF yielded a significantly greater disc angle at L5–S1 than did TLIF (18.4° vs. 6.9°, *p* < 0.001). Proximal junctional kyphosis occurred significantly earlier in the OLIF51 group than in the TLIF51 group (8.6 vs. 26.3 months, *p* < 0.001).

**Conclusion:**

Successful sagittal correction in ASD patients with marked sagittal deformity was achieved with cMIS using OLIF. OLIF at L5–S1 showed a synergistic effect in sagittal deformity correction by cMIS.

## Background

Conventional deformity surgery, including open posterior or combined anterior-posterior approaches, is considered the standard technique for adult spinal deformity (ASD), with reliable clinical and radiological outcomes [1]. However, conventional deformity surgery is known to have a high risk of surgical complications, especially in patients with advanced age [2].

Minimally invasive surgery (MIS) can be an alternative surgical treatment with comparable surgical outcomes and lower complication rates [3–8]. Among various MIS techniques, transforaminal lumbar interbody fusion (TLIF), extreme lateral interbody fusion, or oblique lateral interbody fusion (OLIF) combined with percutaneous posterior spine fixation (PPSF) without any posterior osteotomy are important techniques for minimally invasive deformity [3–6, 8–10]. Deformity correction using MIS techniques without any conventional open surgery is referred to as circumferential MIS (cMIS) [5, 11, 12]. Recently, OLIF at L5–S1 was introduced as a minimally invasive anterior approach and is expected to yield a greater lordotic angle at the L5–S1 level [4, 10]. Although OLIF at L5–S1 is known to effectively improve the lordotic angle, no study has evaluated its effect on deformity correction [4]. Compared with conventional deformity surgery, cMIS is known to be effective for coronal but not for sagittal correction [5–7, 13, 14]. cMIS has been advanced continuously in sagittal correction of ASD [5, 6, 13]. Despite recent advancements, cMIS remains limited by the insufficient correction of marked sagittal deformity [5, 15, 16].

Thus, this study aimed to investigate the effectiveness of cMIS using OLIF for ASD patients with marked sagittal deformity and analyzed the usefulness of OLIF at L5–S1.

## Methods

### Patient population

We retrospectively reviewed the medical records of consecutive patients who underwent surgery for degenerative spinal deformities at a single institute from December 2012 to December 2016. This study was approved by the Institutional Review Board of our hospital (1810-014-16217, 11/19/2018–11/18/2019). We enrolled ASD patients, aged 20 to 80 years old, who preoperatively satisfied the following three factors for sagittal imbalance: sagittal vertical axis (SVA) > 50 mm, pelvic tilt (PT) > 20°, and pelvic incidence to lumbar lordosis mismatch (PI-LL) > 10° [17–19]. We selected 23 patients based on the following inclusion criteria (whole group): (1) marked sagittal deformity preoperatively according to the SRS-Schwab sagittal modifier [19], (2) multilevel MIS lumbar interbody fusion (≥ 4 levels including L5–S1) using OLIF at L1–L5 with TLIF or OLIF at L5–S1, (3) percutaneous fixation (≥ 5 levels) including L5–S1 level, and (4) available follow-up evaluation for at least 24 months after surgery. We defined marked sagittal deformity as the presence of two or more marked sagittal modifiers, SVA > 9.5 cm, PI-LL > 20°, or PT > 30° [19, 20].

The whole group was divided into two groups according to the types of fusion technique at the L5–S1 level: (1) OLIF51 group (*n* = 13), which underwent OLIF at L1–L5 and OLIF at L5–S1 (Fig. 1) and (2) TLIF51 group (*n* = 10), which underwent OLIF at L1–L5 and TLIF at L5–S1 (Fig. 2). All patients received PPSF. The surgical goal was to achieve the following sagittal parameters: SVA < 50 mm, PT < 20°, and PI-LL < 10° [19, 21].

**Fig. 1:**
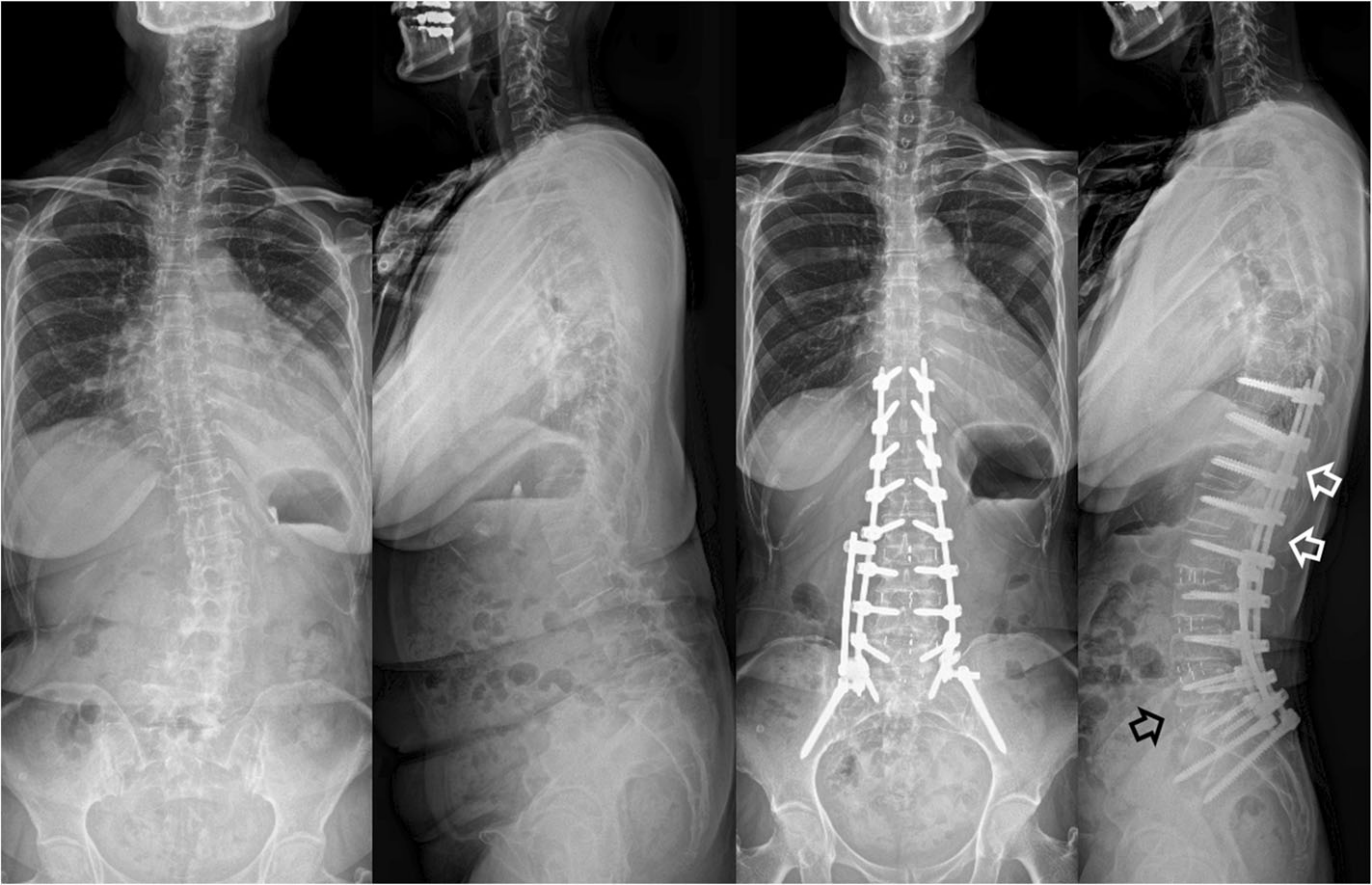
A case in the OLIF51 group: marked sagittal deformity in a 70-year-old woman with adult spinal deformity. She underwent oblique lateral interbody fusion at L1–5 and L5–S1 (black arrow). Posterior fixation was performed with percutaneous pedicle screws and rods system without corrective osteotomy. A lordotic curve was noted at the thoracolumbar junction area (white arrows)

**Fig. 2:**
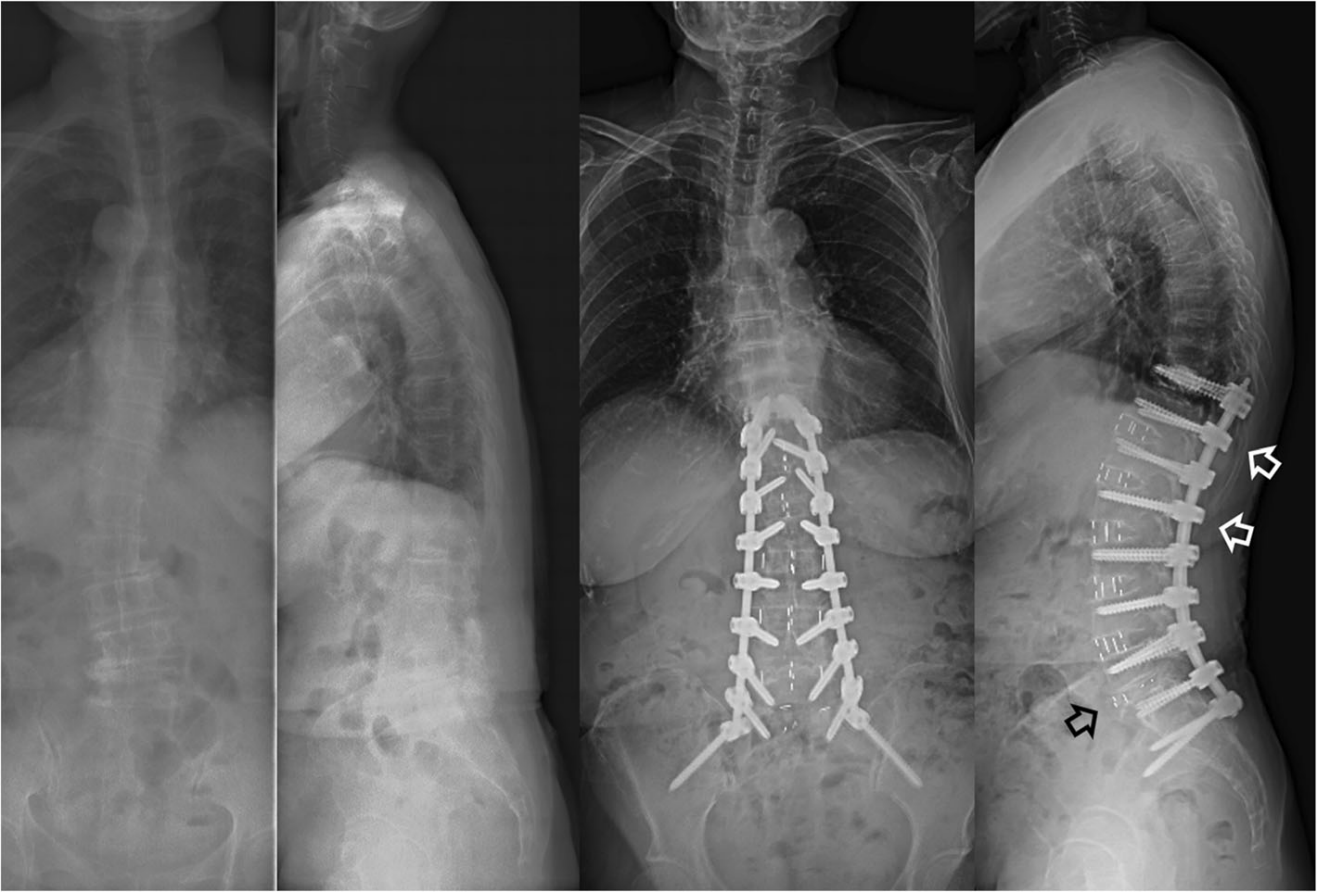
A case in the TLIF51 group: marked sagittal deformity in a 65-year-old woman with adult spinal deformity. The patient underwent direct lateral lumbar interbody fusion at T12–L1, oblique lateral interbody fusion at L1–L5, and transforaminal lumbar interbody fusion at L5–S1 (black arrow). Posterior fixation was performed with percutaneous screws and rods system without corrective osteotomy. A lordotic curve was noted at the thoracolumbar junction area (white arrows)

Patients who had undergone posterior corrective osteotomy or open posterior spine fixation were excluded. Other patients who underwent operation due to tumor, fracture, infection, or congenital anomalies were also excluded.

### Demographic data and clinical outcomes

Demographic data included age, sex, American Society of Anesthesiologists (ASA) score, body mass index (BMI), T-score of bone mineral density (BMD), follow-up period, surgical parameters, and clinical outcomes. Surgical parameters were surgical techniques, number of fusion or fixation levels, operation time, and estimated blood loss (EBL). Clinical outcomes were evaluated with visual analog scale (VAS) score for back or leg pain and Oswestry Disability Index (ODI), which were checked preoperatively and 24 months after surgery.

### OLIF at L1–L5 (Figs. 1 and 2)

We performed OLIF at L1–L5 [10]. Patients were positioned in the right lateral decubitus position without hip flexion. A large rectangular polyether-ether ketone (PEEK) cage (Clydesdale, Medtronic, USA) with 6° or 12° lordotic angle and 20 mm width was inserted into the disc space. We used a 6° cage for the L1–L3 levels and a 12° cage for the L3–L5 levels to make a greater lordotic angle at the lower lumbar levels. The cage was filled with demineralized bone matrix (Grafton, Medtronic, USA) for bone fusion. We tried to insert the cages anteriorly close to the anterior disc margin for more posterior shortening by rod compression.

### OLIF at L5–S1 (Fig. 1)

We performed OLIF at L5–S1 [10]. The patient position was the same as the position of OLIF at L1–L5. We inserted a round-shaped PEEK cage (Perimeter, Medtronic, USA) with 12° lordotic angle into the center of the disc space in an oblique direction. Demineralized bone matrix (Grafton, Medtronic, USA) was also used as fusion material.

### TLIF at L5–S1 (Fig. 2)

TLIF was performed at the L5–S1 level using a tubular retractor system (MAST Quadrant system, Medtronic, USA) in the prone position [9]. We inserted two PEEK cages (Capstone, Medtronic, USA) into the disc space. The cages were packed with local bone chips from the lamina and facets.

### Percutaneous posterior spine fixation

The patient was placed in the prone position with pillows under the chest and pelvis to make a natural lumbar lordotic curve. Posterior fixation was performed percutaneously (CD Horizon Longitude II system, Medtronic, USA) and connected to iliac screws (CD Horizon Legacy system, Medtronic, USA). The iliac screw was inserted through a small opening over the posterior superior iliac spine. Before rod insertion, we bent the motorized operation table to increase lumbar extension. We contoured the rods aggressively to create a greater lordotic curve. Rods were inserted from the iliac screws to the upper instrumented levels percutaneously. Finally, rod compression was done percutaneously for posterior shortening to further increase the lumbar lordotic angle.

### Radiological factors

Various spinopelvic parameters were used for the evaluation of sagittal balance [19, 22]. Accordingly, we checked SVA, PT, PI, and LL at L1–S1; lower lumbar lordosis (LLL) at L4–S1, proportion of LLL in LL (LLL/LL, %), PI-LL, and mean disc angles (DA) at L2–L5 and L5–S1. We checked the postoperative changes in LL (dLL) and SVA (dSVA). All radiological parameters were measured preoperatively and 24 months after surgery. Two observers (SWP and MJK) checked the radiological parameters two times with more than a 2-week interval.

### Complications

Proximal junctional kyphosis (PJK) was assessed as a long-term radiological problem. PJK was defined according to two criteria: (1) the Cobb angle between the upper instrumented vertebra and two-level proximal vertebra was ≥10° and (2) the proximal junctional Cobb angle was 10° or more than the preoperative angle [23]. Other postoperative complications were also evaluated.

### Statistical analysis

The non-parametric Mann-Whitney *U* test and chi-square test were used for comparisons between groups. Preoperative and postoperative VAS scores were compared using the Wilcoxon signed rank test in each group and the Mann-Whitney *U* test between two groups. A value of *p* < 0.05 was considered as statistically significant. The intraobserver and interobserver intraclass correlation coefficients were calculated for the sagittal parameters and DA measured by the two observers [24].

## Results

### Demographic data (Table 1)

Thirty-seven consecutive ASD patients who underwent deformity correction surgery using MIS techniques (OLIF, TLIF, and PPSF) were investigated. Six patients were excluded because they received posterior corrective osteotomies. Three patients were lost to follow-up within 24 months after surgery. Five of 28 patients who underwent cMIS were excluded because their preoperative sagittal parameters did not satisfy the criteria for marked sagittal deformity. Finally, 23 ASD patients who had marked sagittal deformity preoperatively and underwent cMIS were enrolled (whole group). The number of patients in the OLIF51 and TLIF51 groups was 13 and 10, respectively.

**Table 1:** Demographic data

Parameters	Whole group	OLIF51 group	TLIF51 group
No. patients	23	13	10
Age	69.4 ± 5.0	69.8 ± 5.0	68.9 ± 5.3
Female (%)	87.0%	84.6%	90.0%
ASA score	1.9 ± 0.4	1.9 ± 0.3	2.0 ± 0.0
BMI	25.0 ± 3.1	26.0 ± 3.8	23.6 ± 4.8
BMD T-score	− 2.4 ± 0.5	− 2.4 ± 0.6	− 2.5 ± 0.2
Follow-up (months)	40.7 ± 14.6	29.8 ± 4.9***	54.8 ± 9.8
Fusion	OLIF, TLIF	OLIF at L1–S1	OLIF at L1–L5, TLIF at L5–S1
Posterior fixation	Percutaneous	Percutaneous	Percutaneous
No. fusion levels	4.4 ± 0.5	4.4 ± 0.5	4.4 ± 0.5
No. fixation levels	6.7 ± 1.5	6.8 ± 1.5	6.5 ± 1.0
Operation time (min)	345.0 ± 50.0	347.9 ± 60.9	369.0 ± 17.4
EBL (ml)	331.3 ± 109.6	260.7 ± 83.5***	423.0 ± 59.3
Clinical outcomes			
VAS Back			
Before surgery	6.5 ± 1.5	6.4 ± 1.6	6.6 ± 1.5
24 months	2.2 ± 0.6‡	2.1 ± 0.6‡	2.3 ± 0.7‡
VAS leg			
Before surgery	7.4 ± 1.2	7.6 ± 1.1	7.2 ± 1.3
24 months	1.4 ± 0.8‡	1.4 ± 0.8‡	1.3 ± 0.7‡
ODI			
Before surgery	49.9 ± 7.7	49.7 ± 10.2	50.1 ± 4.2
24 months	14.6 ± 3.2‡	14.9 ± 3.3‡	14.2 ± 3.3‡

*ASA score* American Society of Anesthesiologists physical status classification (converted to an Arabic numeral for purposes of analysis), *BMI* body mass index, *BMD* bone mineral density, *OLIF* oblique lateral interbody fusion, *TLIF* transforaminal lumbar interbody fusion, *EBL* estimated blood loss, *VAS* visual analog scale, *ODI* Oswestry disability index

‡*p* < 0.001 compared to before surgery, ***p < 0.001 compared to TLIF51 group

The mean age was 69.6 ± 5.8 years in the whole group. There was no significant difference between the OLIF51 and TLIF51 groups in the mean age and sex ratio.

The mean postoperative follow-up period was 40.7 ± 14.6 (25–69) months in the whole group, and 29.8 ± 4.9 (25–37) and 54.8 ± 9.8 (41–69) months in the OLIF51 and TLIF51 groups, respectively (*p* < 0.001). The significantly longer follow-up period of the TLIF51 group than the OLIF51 group was related with the later start of OLIF at L5–S1 in our hospital.

EBL during the surgery was 331.3 ± 109.6 ml in the whole group, which was significantly lower in the OLIF51 group than in the TLIF51 group (260.7 ± 83.5 ml vs. 423.0 ± 59.3 ml, *p* < 0.001).

The VAS and ODI scores significantly improved postoperatively in the whole group (*p* < 0.001). There was no significant difference in the pre- and postoperative VAS and ODI scores between the OLIF51 and TLIF51 groups.

### Radiological parameters (Table 2)

The preoperative SVA, PT, LL, and PI-LL were 125.7 ± 21.1 mm, 33.0 ± 7.2°, 18.2 ± 9.0°, and 35.5 ± 9.4° in the whole group. The preoperative radiological parameters were not significantly different between the OLIF51 and TLIF51 groups.

**Table 2:** Radiological parameters

	Before surgery				24 months after surgery	
	Whole	OLIF51	TLIF51	Whole	OLIF51	TLIF51
SVA (mm)	125.7 ± 21.1	125.9 ± 21.3	125.5 ± 22.1	29.5 ± 14.8^‡^	27.1 ± 11.4	32.7 ± 18.4
PT (°)	33.0 ± 7.2	31.4 ± 7.2	35.1 ± 7.0	18.1 ± 5.7^‡^	17.6 ± 4.6	18.6 ± 7.6
PI (°)	53.7 ± 6.4	53.2 ± 6.0	54.4 ± 7.1	53.6 ± 6.5	53.0 ± 6.0	54.4 ± 7.2
LL (°)	18.2 ± 9.0	16.7 ± 10.4	20.3 ± 6.8	51.7 ± 5.8^‡‡^ (39.4–62.4)	55.5 ± 2.8*** (50.0–62.4)	46.9 ± 5.2 (39.4–52.6)
LLL (°)	9.5 ± 4.0	9.0 ± 4.6	10.2 ± 3.3	27.2 ± 4.8^‡‡^	31.1 ± 1.4***	22.3 ± 2.4
LLL/LL (%)	49.4 ± 17.4	47.5 ± 18.2	51.9 ± 17.0	52.5 ± 5.5	56.2 ± 2.1***	47.8 ± 4.9
PI-LL (°)	35.5 ± 9.4	36.5 ± 8.5	34.1 ± 10.6	5.3 ± 3.6^‡‡^ (1.0–10.6)	3.6 ± 3.0** (1.0–10.6)	7.5 ± 3.2 (3.5–10.0)
dSVA (mm)	–	–	–	96.2 ± 23.4 (55.3–141.5)	98.9 ± 22.9	92.8 ± 24.8
dLL (°)	–	–	–	33.4 ± 11.5 (10.0–67.5)	38.7 ± 10.2** (32.3–67.5)	26.6 ± 9.8 (10.0–39.9)
Mean DA (°)						
L2-L5	4.1 ± 3.5	3.4 ± 4.1	5.0 ± 2.4	12.4 ± 1.5^‡‡^ (8.1–16.7)	12.5 ± 1.6	12.3 ± 1.4
L5-S1	8.7 ± 3.5	9.8 ± 3.6	7.5 ± 2.3	12.9 ± 6.7^‡^ (3.5–27.9)	18.4 ± 3.7*** (14.5–27.9)	6.9 ± 2.8 (3.5–10.7)

*OLIF* oblique lateral interbody fusion, *TLIF* transforaminal lumbar interbody fusion, *SVA* sagittal vertical axis, *TK* thoracic kyphosis, *PT* pelvic tilt, *PI* pelvic incidence, *LL* lumbar lordosis at L1–S1, *LLL* lumbar lordosis at L4–S1, *LLL/LL* LLL/LL × 100 (%), *dSVA* difference between pre- and postoperative values of SVA, *dLL* difference between pre- and postoperative values of LL, *DA* disc angle

‡*p* < 0.01 and ‡‡*p* < 0.001 compared to before surgery, **p < 0.01 and ***p < 0.001 compared to TLIF51 group

No significant difference in the preoperative values between the TLIF51 and OLIF51 groups

In the whole group, the SVA (29.5 ± 14.8 mm, *p* < 0.001), PT (18.1 ± 5.7°, *p* < 0.01), LL (51.7 ± 5.8°, *p* < 0.001), and PI-LL (5.3 ± 3.6°, p < 0.001) significantly improved postoperatively. Additionally, the OLIF51 group showed significantly higher LL (55.5 ± 2.8° vs. 46.9 ± 5.2°, *p* < 0.001) and significantly lower PI-LL (3.6 ± 3.0° vs. 7.5 ± 3.2°, *p* < 0.01) than the TLIF51 group.

Postoperative LLL at L4–S1 was greater in the OLIF51 than in the TLIF51 group (31.1 ± 1.4° vs. 22.3 ± 2.4°, *p* < 0.001). Hence, the postoperative proportion of LLL in the total LL (LLL/LL) was significantly greater in the OLIF51 than in the TLIF51 group (56.2 ± 2.1% vs. 47.8 ± 4.9%, *p* < 0.001).

In the whole group, dSVA and dLL were 96.2 ± 23.4 mm (55.3–141.5) and 33.4 ± 11.5° (10.0–67.5), respectively. dLL was significantly greater in the OLIF51 than in the TLIF51 group (38.7 ± 10.2° vs. 26.6 ± 9.8°, *p* < 0.01).

The mean DA at L2–5 (4.1 ± 3.5° vs. 12.4 ± 1.5°, *p* < 0.001) and L5–S1 (8.7 ± 3.5° vs. 12.9 ± 6.7°, *p* < 0.01) significantly improved postoperatively in the whole group. Although there was no significant difference in the postoperative DA at L2–L5, postoperative DA at L5–S1 was significantly higher in the OLIF51 than in the TLIF51 group (18.4 ± 3.7° vs. 6.9 ± 2.8°, *p* < 0.001).

### Complications

The incidence of PJK was 30.4% in the whole group; similar values were found in the OLIF51 and TLIF51 groups (31.0% and 30.0%). However, PJK occurred significantly earlier postoperatively in the OLIF51 than in the TLIF51 group (8.6 ± 1.9 vs. 26.3 ± 4.7 months, *p* < 0.001, Table 3).

**Table 3:** Proximal junctional kyphosis and rod fracture

	Whole group	OLIF51 group	TLIF51 group
Number of PJK	7 (30.4%)	4 (31.0%)	3 (30.0%)
Onset (postop months)	16.1 ± 10.0	8.6 ± 1.9 (7–10)***	26.3 ± 4.7 (21–30)
Cause of PJK			
Adjacent fracture	7	4	3
Screw loosening	3	2	1
Management			
Revision surgery	2	1	1
Vertebroplasty	4	2	2

*PJK* proximal junctional kyphosis

***p < 0.001 compared to TLIF51 group

Psoas symptoms (8/23), ileus (13/23), and leg dysesthesia (5/23) were noted postoperatively. Postoperative ileus occurred more frequently in the OLIF51 (10/13) than in the TLIF51 group (3/10) (*p* < 0.05). There were no major complications.

### Intraobserver and interobserver reliabilities

Both intraobserver and interobserver reliabilities were within acceptable ranges (0.85–0.92 and 0.75–0.83, respectively).

## Discussion

The recent concept of cMIS is a combination of MIS lumbar interbody fusion and percutaneous fixation without osteotomy [3, 5–8]. Previously, cMIS was not indicated for sagittal correction of ASD [7, 14, 25, 26]. Recent studies reported that cMIS could be indicated only for mild deformity, but osteotomies were required for marked deformity [4, 5]. However, according to our study, cMIS also seems to be effective for sagittal correction even in patients with marked deformity.

A ceiling effect is referred to as the maximum correction of sagittal parameters allowed by deformity surgery [16, 26]. Anand et al. reported that the ceiling effect of dSVA was 120 mm for cMIS [16]. In our study, the maximum dSVA was 141.5 mm, which was greater than that previously reported. Other studies reported the maximum dLL and corrected LL by cMIS were 23° and 54°, respectively [25, 26]. In our study, the maximum dLL and corrected LL were 67.5° and 62.4°, respectively, which were also greater than those of previous reports. Due to the increased angle correction, the postoperative sagittal parameters satisfied the surgical goals in our study. The greater LL correction in our study seemed to be related to the combination of OLIF with high-angle cages placed anteriorly in the disc space, PPSF with aggressively contoured rods, and intraoperative lumbar extension with a motorized operation table followed by percutaneous rod compression. Our study is the first to show successful correction of marked sagittal deformity with cMIS in ASD.

The mean postoperative LL was 51.7° in the whole group, which might suffice for most of the Korean population whose mean PI is relatively lower (47.8°) than the western population (50.2°–52.0°) [27–30]. The mean postoperative LL was greater in the OLIF51 than in the TLIF51 group, while the mean postoperative LL in the TLIF51 group was similar to those of other studies using DLIF at lumbar levels and TLIF or AxiaLIF at L5–S1 [12, 25]. The greater mean postoperative LL in the OLIF51 group than in the TLIF51 group seemed to be due to the significant increase in disc angle at the L5–S1 level because the L5–S1 level contributes largely to the lumbar lordosis [31]. During OLIF at L5–S1, the anterior disc space was opened widely as in anterior lumbar interbody fusion (ALIF), which helped to create a larger disc angle than that with TLIF [32–34]. Since TLIF was introduced in 1982, it was developed as a MIS technique [12, 33]. We had been mainly used TLIF at L5–S1 level before the introduction of OLIF51. Bilateral facetectomy and TLIF with banana-shaped cages were previously reported to increase segmental angles [35, 36]. However, there may be some debates because other studies observed no significant improvement in segmental angles after TLIF and no difference according to the TLIF cage type [32, 33].

Hybrid surgery is known to be more effective than cMIS in sagittal correction, with the drawbacks of increased operating time and EBL [7, 12, 25, 37]. However, the sagittal correction, operating time, and EBL in our study were better than those in other cMIS and hybrid surgery studies even though our patients had worse sagittal deformity preoperatively (Table 4). These better results seem to be related to OLIF use at L5–S1, given the improved results in the OLIF51 than in the TLIF51 group. TLIF and axial lumbar interbody fusion (AxiaLIF) for cMIS or ALIF for the hybrid surgery were the fusion techniques used at L5–S1 previously. Our study is also the first report evaluating the effects of OLIF at L5–S1 on sagittal deformity correction.

**Table 4:** Summary of our data and literature using lateral interbody fusion for correction of adult spinal deformity

	Wang et al. [25]	Haque et al. [37]	Wang et al. [26]	Park et al. [12]	Theologis et al. [7]	Our study
No. patients	23	48	43	43	16	23
Deformity surgery	cMIS	cMIS	cMIS	cMIS	Hybrid	cMIS
Fusion at L1–5	DLIF, TLIF	LLIF, TLIF, ALIF	XLIF, TLIF	DLIF, TLIF	LLIF	OLIF
Fusion at L5–S1	TLIF	NS	TLIF	AxiaLIF	TLIF, ALIF	TLIF, OLIF
Posterior fixation	PPSF	PPSF	PPSF	PPSF	OPSF	PPSF
Fusion levels	3.7 ± 1.3	4.7 ± 2.8	3–7	4.0 ± 1.1	4.6 ± 1.1	4.4 ± 0.5
Operating time (min)	402.0 ± 122.3	462.0 ± 177.0	479	452.4 ± 212.2	859.1 ± 194.8	345.0 ± 50.0
EBL (ml)	477.4 ± 673.5	507.0 ± 841.0	585	552.4 ± 460.1	2460.0 ± 1405.2	331.3 ± 109.6
Preoperative						
SVA (mm)	NR	33.0	29.0 ± 41.4	30.0 ± 54.1	62.7 ± 50.8	125.7 ± 21.1
LL (°)	37.4	33.8	34.0 ± 10.9	41.2 ± 15.6	26.6 ± 22.6	18.2 ± 9.0
PI-LL	NR	21.6	NR	10.2 ± 15.6	23.4 ± 22.0	35.5 ± 9.4
Postoperative						
SVA	NR	32.7	29.7 ± 44.6	32.1 ± 70.1	35.8 ± 41.9	29.5 ± 14.8
dSVA	NR	− 0.3	− 0.7	− 2.1	26.9	96.2 ± 23.4
LL	45.5	39.4	39.6 ± 12.1	44.1 ± 13.1	43.2 ± 15.6	51.7 ± 5.8
dLL	12.1	5.6	5.6	2.9	16.6	33.4 ± 11.5
PI-LL	NR	16.0	NR	8.0 ± 14.4	7.4 ± 12.4	5.3 ± 3.6

*cMIS* circumferential minimally invasive surgery, *Hybrid* MIS + open surgery, *DLIF* direct lateral interbody fusion, *XLIF* extreme lateral interbody fusion, *TLIF* transforaminal lumbar interbody fusion, *ALIF* anterior lumbar interbody fusion, *AxiaLIF* axial lumbar interbody fusion, *OLIF* oblique lateral interbody fusion, *PPSF* percutaneous posterior spine fusion, *OPSF* open posterior spine fusion, *EBL* estimated blood loss, *PI* pelvic incidence, *LL* lumbar lordosis at L1–S1, *dSVA* difference between pre- and postoperative values of SVA, *dLL* difference between pre- and postoperative values of LL, *NS* not specified, *NR* not reported

Significantly earlier PJK onset was observed in the OLIF51 group (8.6 months) than in the TLIF51 group (26.3 months), which was also earlier than that of previous reports (18.6–34.8 months) [38, 39]. Other reports found that the incidence of PJK could be reduced by minimally invasive lumbar interbody fusion and percutaneous fixation, which suggests that other factors may have accelerated PJK development after cMIS with OLIF at L5–S1 in our study [40, 41]. Faundez et al. reported an exaggerated upper lumbar lordosis resulting in junctional failure [39]. Previous studies reported that the ideal LLL/LL was 50–80% [30, 42]. According to our data, the LLL/LL of the OLIF51 group was within the ideal range. However, there was a lordotic curve at the thoracolumbar junction, which showed similar effect of exaggerated upper lumbar lordosis. The lordotic angle at the thoracolumbar junction related with the lordotic curve of the proximal rod (Fig. 1). The lordotic curve of the proximal rod was necessary to pass a long rod percutaneously through the lumbosacral curvature. However, it resulted in increased lordosis at the thoracolumbar junction, which might lead to early PJK. Postoperatively smaller SVA, higher LL correction, and smaller PI-LL in the OLIF51 group also seemed to contribute to earlier PJK onset [38, 43].

The retrospective design is the major limitation of this study. Although the follow-up period was at least 2 years, a larger number of patients and longer follow-up is necessary because correcting marked sagittal deformity using cMIS is a relatively new trial. Multicenter studies performing similar cMIS are therefore needed. We suggested several factors responsible for the improved correction of marked sagittal deformity, but other mechanisms should be further investigated. Rigid deformity itself was not evaluated in this study, but we could not rule out completely the effect of rigid joints on the sagittal correction by cMIS. Considering that early PJK in the OLIF51 group is associated with exaggerated proximal lordosis, we tried to reduce it by making a kyphotic angle at the proximal rods (data not presented), but still do not know the exact underlying cause because the preventive effects can only be observed after a longer period. Our results cannot be applied for adolescent idiopathic scoliosis because we studied only for ASD.

## Conclusions

According to our data, the use of OLIF with cMIS seemed to help to overcome the main limitation of cMIS in correcting marked sagittal deformity of ASD. Although OLIF at L5–S1 showed a synergistic effect on the sagittal correction with cMIS, early PJK remained an unsolved issue of cMIS using OLIF at L5–S1.

## Data Availability

The datasets used during the current study are available from the corresponding author upon reasonable request.

## Abbreviations

ASA: American Society of Anesthesiologists
ASD: Adult spinal deformity
BMD: Bone mineral density
BMI: Body mass index
cMIS: Circumferential minimally invasive surgery
DA: Disc angle
LL: Lumbar lordosis
LLL: Lower lumbar lordosis
ODI: Oswestry Disability Index
OLIF: Oblique lateral interbody fusion
PEEK: Polyether-ether ketone
PI: Pelvic incidence
PJK: Proximal junctional kyphosis
PPSF: Percutaneous posterior spine fixation
PT: Pelvic tilt SVA Sagittal vertebral axis
TLIF: Transforaminal lumbar interbody fusion
VAS: Visual analog scale
